# A pilot cluster-randomized controlled trial of an audit, feedback, and coaching intervention on compliance with elementary physical education laws and student physical activity during lesson time

**DOI:** 10.1016/j.pmedr.2025.103037

**Published:** 2025-03-19

**Authors:** Hannah R. Thompson, Caroline Nguyen, Thomas L. McKenzie, David A. Dzewaltowski, Kristine A. Madsen

**Affiliations:** a Community Health Sciences, School of Public Health, UC Berkeley, Berkeley, CA, United States; b School of Exercise and Nutritional Sciences, San Diego State University, San Diego, CA, United States; c Department of Health Promotion, College of Public Health, University of Nebraska Medical Center, Omaha, NE, United States

**Keywords:** Physical education, Health law compliance, Moderate-to-vigorous physical activity, School health

## Abstract

**Objective:**

Compliance with elementary school physical education law is low. School district-led PE audit, feedback, and coaching (PEAFC), along with funding credentialed teachers, demonstrated the potential for improving compliance with law in New York City public schools. However, the likely scalable approach of PEAFC, alone, has not been rigorously tested in other districts.

**Methods:**

Two-year pilot cluster-RCT in 10 Bay Area, California elementary schools (mean enrollment 421; 66 % Latino; 92 % free or reduced-price meal eligible). Five schools were randomized to receive PEAFC. Physical education lessons (*n* = 168) were observed using the System for Observing Fitness Instruction Time in Fall 2022, Spring 2023, and Spring 2024. Linear mixed effects models examined the impact of PEAFC on between-group changes in law compliance (using scheduled and estimated physical education minutes) and lesson time in moderate-to-vigorous physical activity (MVPA).

**Results:**

There were no statistically significant differences in changes in scheduled or estimated minutes between times between groups. Students in both intervention (10 % increase; 95 % CI: 2.17 %, 17.41 %) and control (9 % increase; 95 % CI: 2.61 %, 14.42 %) schools increased lesson time in MVPA, but there was not a statistically significant difference in change between groups.

**Discussion:**

PEAFC did not impact physical education law compliance or lesson time spent in MVPA. In the absence of credentialed physical education teachers to provide mandated minutes, PEAFC may be limited in its potential to increase compliance with state mandates. Hiring credentialed physical education teachers in elementary schools may be the most effective way to support compliance with state physical education laws.

## Highlights

• Compliance with state-level elementary school physical education law is low.
• We tested physical education audit, feedback, and coaching on law compliance.
• In this randomized trial, the intervention did not impact law compliance.
• Without credentialed physical education teachers, the intervention was limited.
• Funding credentialed teachers may be most effective for increasing law compliance.

## 1 Introduction

School physical education is an important public health tool for improving youth cardiorespiratory fitness and supporting the attainment of the recommended 60 min of daily moderate-to-vigorous physical activity (MVPA) (*U.S. Department of Health and Human Services. Physical Activity Guidelines for Americans, 2nd edition. Washington, DC: U.S. Department of Health and Human Services; 2018. Available at*, 2025; Thompson et al., 2019a; *Institute of Medicine. Educating the Student Body: Taking Physical Activity and Physical Education to School. Concensus Report. May 2013. Available at*, 2025).

As of 2022, all but one US state had a law requiring physical education, with 24 % of states requiring at least 60 min of physical education/week in elementary schools (to ensure sufficient physical education *quantity*) and 24 % requiring physical education certification/ licensure to teach the subject at the elementary level (to help ensure higher class *quality*) (*National Institute of Health. National Cancer Institute. Classification of Laws Associated with School Students. Physical Education 2003-2022 Data File. Available at*, 2025). Multiple studies have demonstrated students spend more time in standards-based activities and in health-enhancing MVPA when physical education is taught by a credentialed physical education teacher (Thompson et al., 2019a; McKenzie et al., 2001; Shape America and the American Heart Association, 2016; Carlson et al., 2013). While the high prevalence of physical education laws demonstrates the subject's value, compliance with physical education laws is often low due to competing school priorities and a lack of funding for physical education teachers (Thompson et al., 2019a; Thompson et al., 2015a; Thompson et al., 2018). Elementary schools are the least compliant with physical education laws (Shape America and the American Heart Association, 2016), and are thus a key target for interventions that increase law compliance.

Extensive research in California has shown that a lack of accountability is a major factor in low elementary physical education law compliance (Thompson et al., 2015a; Thompson et al., 2018; Thompson et al., 2015b). However, means of holding schools accountable for physical education are inconsistent (or non-existent) across states and best practices remain unknown. Research suggests audit and feedback could be an effective mechanism for improving physical education implementation and adherence with state law; collecting and publicly sharing data from 20 San Francisco elementary schools increased physical education quantity by an average of 14 min/week (25 % relative increase) and led to a 7 % increase in in-class MVPA (quality) after two years (Thompson et al., 2015a). Additional school-based observational evidence suggests that audit and feedback systems can support curriculum planning (Schildkamp and Visscher, 2010) and improve teaching quality (Schildkamp and Visscher, 2009) and student academic outcomes (Hammond and Yeshanew, 2007). However, the impact of audit and feedback on physical education quantity and quality remains understudied.

To address low elementary physical education law implementation, the New York City Department of Education (NYCDOE) implemented PE Works from school year 2015/16 through 2018/19, which provided schools with physical education teachers and included a district-led audit of school physical education law implementation, along with feedback and coaching with principals and physical education teachers. Qualitative findings from NYC's natural experiment suggested that physical education audits, feedback, and coaching (PEAFC) were a critical piece of PE Works that supported schools in establishing long-term plans for successfully implementing physical education law (Thompson et al., 2023). Given that hiring physical education teachers may be cost-prohibitive in many districts, testing PEAFC to improve physical education law compliance in other districts is warranted.

The purpose of this two-year pilot cluster randomized trial was to determine the impact of PEAFC (adapted from lessons learned in NYCDOE) on physical education law compliance (quantity) and the proportion of physical education lesson time spent in MVPA (quality) in public elementary schools in an urban Bay Area California school district.

## 2 Methods

### 2.1 Setting and participants

This two-year (2022/23–2023/24) cluster-randomized controlled trial involved 10 elementary schools conducted in partnership with the district's teacher on special assignment for physical education (hereafter, “Physical Education Director”). Study schools were chosen using a survey of school principals which identified schools that were non-compliant (or unsure about compliance) with California law mandating 200 min of physical education every 10 school days (Thompson et al., 2019b). Schools were randomized to intervention (*n* = 5) and control (n = 5) arms using block randomization, ensuring equal representation by total enrollment and proportion of low-income students enrolled (Sanchez-Vaznaugh et al., 2012) across groups. Schools received $1000/year for participation. The University of California Berkeley Committee for the Protection of Human Subjects (#2020–09-13,643) and the school district's Institutional Review Board approved all study measures. This study is registered at ClinicalTrials.gov (NCT05509803).

### 2.2 PEAFC tool

The PEAFC tool was adapted from NYCDOE's tool using: 1) findings from NYCDOE (Thompson et al., 2023); 2) data collected through semi-structured interviews with 20 principals from non-study schools on potential best-practices for PEAFC; and 3) in collaboration with the district's Physical Education Director. It consisted of five yes/no indicators of physical education quantity and quality indicators: 1) Schedule all students for physical education that meets California time requirements of the equivalent of 100 min physical education/week; 2) Hire credentialed physical education teachers and assign them with appropriate student-to-staff ratios; 3) Encourage and support teachers in participating in professional development learning opportunities; 4) Observe instruction to ensure physical education content standards and grade-level outcomes; 5) Educate school community, including teachers and parents, about physical activity and other wellness opportunities.

The Physical Education Director was trained to implement PEAFC in the summer prior to Year 1 and collected audit data during Fall of Year 1 through visits to each intervention school. During these visits, he spoke with principals, physical education teachers, and classroom teachers, as well as observed physical education classes, to determine which of, and to what extent, the five audit indicators were being met. The Physical Education Director gave feedback on the audit findings during Fall Year 1 (after all pre-intervention data collection, to ensure a true baseline) in face-to-face meetings with the physical education teachers in which they worked on an action plan for improvements. For example, if a physical education teacher was not using a structured curriculum that met California state physical education model content standards, the Physical Education Director worked with the physical education teacher to identify a curriculum, as well as a plan for implementation. Of note, the intervention protocol also included meeting with school principals to provide feedback on the audit results, most specifically to discuss physical education quantity, to address adherence/non-adherence to state physical education minute law. However, due to limited capacity with his other district duties, he was not able to do so. From Spring Year 1 and Fall/Spring Year 2 the Physical Education director provided ongoing coaching for physical education teachers, which ranged from setting up organizational systems for physical education equipment to curriculum, pedagogy, and behavior management to helping identify appropriate professional development opportunities.

### 2.3 Physical education lesson observations

Data collection took place in Fall (September–November) 2022 (pre-intervention), Spring (February–May) 2023 (Year 1), and Spring 2024 (Year 2) using the validated System for Observing Fitness Instruction Time (SOFIT) (McKenzie, 2015). At each timepoint, researchers obtained physical education master schedules, which contained physical education times for all classes taught at the school. In addition, they collected individual classroom physical education schedules for all second/fifth grade teachers (to determine if additional physical education was being taught that was not on the master schedule) (Singh et al., 2015). Second and fifth grades were chosen because physical education often operates differently in lower (kindergarten – second) and upper (third - fifth) elementary grades (Singh et al., 2015). These schedules were used to determine the scheduled minutes of physical education/week for second and fifth grade classes across each school. For example, if there were two second grade classes and three fifth grade classes at one school, with scheduled minutes of physical education of 50 and 60 min/week for second grade classes (average 55 mins/week) and 50, 60, and 60 min/week for fifth grade classes (average 57 mins/week for fifth grade), the average minutes of scheduled physical education/week at that school was 56. The physical education schedules were also used to determine who was teaching physical education (credentialed physical education teacher; classroom teacher; or other teacher (e.g. dance teacher, non-credentialed physical education teacher)).

At each timepoint, at each study school, a trained researcher used SOFIT to observe 6 total physical education lessons on randomly selected unique days - three observations each for second and fifth grades. Random day observation enabled verification of whether physical education occurred as scheduled. The observed lesson length (the number of minutes that physical education actually occurred, with observations beginning when 50 % of students had entered the physical education area and stopping at the class's termination) was recorded. Estimated minutes were calculated as observed physical education lesson length times show rate. For example, if a teacher had one 50-min physical education lesson scheduled per week, the lesson was observed to only be 45 min, and the observed teacher had a show-rate of 75 % (only 3/4 lessons observed occurred as scheduled), the estimated minutes of physical education/week would be 34.

### 2.4 Statistical analysis

School-level demographic data were downloaded from the California Department of Education (*California Department of Education. Dataquest State Education Data Reporting. Available at*, 2025). Differences in school-level characteristics between all district elementary schools and study schools, as well as between intervention and control schools, were determined using unpaired *t*-tests. Linear mixed effects models with random effects for school and grade were used to determine 1) within-group changes and 2) difference in change between intervention and control schools in: scheduled minutes of physical education/week; estimated minutes of physical education/week; and the proportion of observed lesson time in MVPA. All analyses were performed using Stata/SE (16.1).

## 3 Results

Pre-intervention, study schools had a mean enrollment of 421 students, 66 % Latino and 92 % who qualified for free or reduced-price meals (FRPM; a proxy for socioeconomic status; Table 1). Study schools had a higher Latino (*p* = 0.01) and FRPM (*p* = 0.04) enrollment compared with elementary schools across the district. Two intervention and three control schools had a full-time credentialed physical education teacher during both study years. At schools without a full-time credentialed physical education teacher, physical education was taught by a non-credentialed physical education teacher (*n* = 1 intervention, 1 control), a part-time credentialed physical education teacher (n = 1 intervention) or a dance teacher (n = 1 intervention, 1 control). physical education lessons were not taught by second or fifth grade classroom teachers at any of the 10 study schools.

**Table 1:** Baseline (school year 2022/23) demographic characteristics of Bay Area, California district (*n* = 62) and study (*n* = 10) elementary schools.

	All district elementary schools (n = 62)	All study schools (n = 10)	p-value for difference between all district and all study schools^A^	Intervention schools (*n* = 5)	Control schools (n = 5)	p-value for difference between intervention and control schools^A^
Total enrollment, mean ± SD	399 ± 146.4	421 ± 151.3	0.612	336 ± 68	505 ± 171	0.07
Asian enrollment, % ± SD	7.1 ± 12.8	3.0 ± 6.3	0.276	1.3 ± 2.9	4.7 ± 8.6	0.42
African American enrollment, % ± SD	22.0 ± 16.0	16.7 ± 11.1	0.261	18.2 ± 15.2	15.2 ± 6.2	0.69
Latino enrollment, % ± SD	46.1 ± 28.0	66.4 ± 23.6	0.011	62.4 ± 30.9	70.5 ± 16.1	0.62
White enrollment, % ± SD	11.0 ± 13.4	5.8 ± 7.4	0.180	8.6 ± 9.5	3.3 ± 3.5	0.26
Multiple races/other race enrollment, % ± SD	9.1 ± 9.6	4.6 ± 5.9	0.105	6.3 ± 7.8	2.9 ± 3.4	0.40
Free or reduced-price meal eligible enrollment, % ± SD	76.2 ± 26.1	92.0 ± 11.0	0.035	88.4 ± 15.0	95.6 ± 3.6	0.33

A *p*-values for differences were calculated using unpaired *t*-tests.

Physical education lessons (*n* = 168 total) were observed pre-intervention (*n* = 24 intervention, 27 control), Year 1 (*n* = 28 intervention, 32 control), and Year 2 (*n* = 30 intervention, 27 control; Table 2). According to schools' master schedules, pre-intervention, three schools (2 intervention, 1 control) met the state physical education mandate equivalent of 100 mins physical education/week. Schools with a physical education teacher had an average 80.0 ± 25.8 scheduled physical education minutes/week pre-intervention, compared with 44.4 ± 25.5 scheduled minutes/week at schools without a physical education teacher. At Year 2, only two schools (1 intervention, 1 control) met the mandate. At baseline, all three schools that met the physical education mandate had full-time credentialed physical education teachers. The majority (*n* = 5; 71 %) of the schools that did not meet the mandate did not have full-time credentialed physical education teachers.

**Table 2:** School year 2022/23–2023/24 changes in scheduled and estimated weekly minutes of physical education and proportion of lesson time spent in moderate-to-vigorous physical activity between Bay Area, California elementary intervention and control schools (n = 10).

	Intervention Schools (n = 5)				Control Schools (n = 5)				
	Pre-intervention, Fall 2022 (n_obs_^A^=24)	Year 1, Spring 2023 (n_obs_ = 28)	Year 2, Spring 2024 (n_obs_ = 30)	Change Pre-Intervention to Year 2^B^ (95 % CI)	Pre-intervention, Fall 2022 (n_obs_ = 27)	Year 1, Spring 2023 (n_obs_ = 32)	Year 2, Spring 2024 (n_obs_ = 27)	Change Pre-Intervention to Year 2^B^ (95 % CI)	Difference in change pre-intervention to Year 2 between Intervention and Control Schools^C^ (95 % CI)
Scheduled minutes of Physical education/week, mean ± SD	60.0 ± 39.4	59.0 ± 21.7	56 ± 24.6	−4.0 ± 6.5 (−16.82, 8.82)	63.8 ± 20.0	62.0 ± 20.4	65.3 ± 19.1	2.3 ± 1.8 (−1.13, 5.75)	−6.3 ± 7.1 (−20.17, 7.52)
Estimated^D^ minutes of Physical education/week, mean ± SD	55.0 ± 34.9	51.6 ± 23.7	53.8 ± 27.5	−1.3 ± 6.0 (−13.08, 10.58)	54.9 ± 22.6	47.0 ± 28.9	48.4 ± 10.4	−8.0 ± 7.2 (−22.05, 6.01)	7.3 ± 9.4 (−11.02, 25.68)
% of lesson time in moderate-to-vigorous physical activity, % ± SD	35.4 ± 19.6	41.4 ± 13.5	45.2 ± 16.6	9.8 ± 3.9 (2.17, 17.42)	43.2 ± 11.5	46.7 ± 13.1	51.3 ± 13.1	8.5 ± 3.0 (2.61, 14.42)	1.3 ± 5.0 (−8.423, 11.11)

A Number of second and fifth grade physical education lesson observations using the validated System for Observing Fitness Instruction (SOFIT) time.

B Estimated using linear mixed effects models with random effects for school and grade.

C Estimated using linear mixed effects models with a group by time interaction term and random effects for school and grade.

D Takes into account proportion of lessons that occurred based on SOFIT observations and the proportion of no-shows (times observer went to observe a randomly selected lesson and the lesson did not occur).

Pre-intervention, intervention schools had an estimated 55 of 60 scheduled minutes of physical education/week occurring; control schools had an estimated 55 of 64 scheduled minutes of physical education/week occurring. At Year 2, intervention schools had an estimated 54 of 56 scheduled minutes of physical education/week occurring; control schools had an estimated 48 of 65 scheduled minutes physical education/week occurring. There was no statistically significant difference in the change in scheduled lessons or estimated physical education minutes between times between groups.

Pre-intervention, students in intervention schools spent an average of 35 % of lesson time in MVPA and increased to 45 % (10 % difference; 95 % CI: 2.17 %, 17.42 %) at Year 2. Pre-intervention, control students spent an average of 43 % of lesson time in MVPA and increased to 51 % (9 % difference; 95 %CI: 2.61 %, 14.42 %) at Year 2. There was no statistically significant difference in the change in proportion of lesson time in MVPA between times between groups.

## 4 Discussion

In this pilot cluster randomized trial in 10 low-income elementary schools, PEAFC did not have an impact on objective measures of physical education minute law compliance or lesson time spent in MVPA. These results add to existing evidence demonstrating the challenges of increasing compliance with physical education law without investing in more costly resources (e.g. funding for physical education teachers or training for classroom teachers to teach physical education) (Thompson et al., 2015a; Thompson et al., 2018; Thompson et al., 2023).

While using PEAFC in the absence of funding to hire/support new physical education teachers was hypothesized to drive improvements in meeting physical education mandates, the present study found no increase in physical education minutes with PEAFC alone. Notably, the number of physical education teachers (range 0–1) at each school was insufficient to deliver 100 min physical education/week to every student at most study schools. Without hiring more physical education teachers, PEAFC was limited in its impact on physical education quantity. Prior evidence demonstrates the perceived value (Thompson et al., 2018; Lounsbery et al., 2019); the cardiorespiratory benefits for students (Thompson et al., 2019a; Carlson et al., 2013; Telford et al., 2016); and schools' increased likelihood of physical education law compliance (Turner et al., 2017) with a credentialed elementary physical education teacher(s) on staff. At baseline, all three schools (100 %) that met the state physical education mandate had full-time credentialed physical education teachers; the majority (71 %) of the schools that did not meet the mandate did not have full-time credentialed physical education teachers. Further, classroom teachers did not offer physical education in any of the study schools. Educational organizations recommend, and it is common practice for, elementary classroom teachers to supplement physical education lessons in the absence of a physical education teacher or when the physical education teacher-to-student ratio is too high to enable the physical education teacher to provide mandated physical education minutes to all students (Thompson et al., 2019a; California School Board Association, 2025; *California Department of Education. Physical education FAQs. Available at*, 2025). No classroom teachers in any of the study schools taught physical education, which also contributed to the low levels of physical education minute law compliance.

Evidence from San Francisco on the success of collecting and disclosing local physical education data on physical education law compliance (Thompson et al., 2015a), coupled with qualitative data from NYCDOE highlighting the perceived value of PEAFC for improving physical education programs (Thompson et al., 2023), provided strong rationale for pursuing the present line of inquiry. However, while the current study was underway, quantitative evidence modeling singular physical education component impacts in NYCDOE demonstrated that hiring a physical education teacher had a significant positive impact on student cardiorespiratory fitness, whereas PEAFC had none (Thompson et al., 2024), further reinforcing the present study's findings.

Increasing compliance with state physical education law is challenging in the face of limited funding (Thompson et al., 2015a; Thompson et al., 2018; Thompson et al., 2023). The Physical Education Director was responsible for all 84 district schools (including middle and high schools), giving him limited time to work directly with intervention schools, which led to low intervention implementation fidelity (primarily in regards to feedback and coaching with principals). When PEAFC was successfully delivered in NYCDOE, teams of two district administrators (one with an administrative background and one with a physical education background) delivered the feedback and coaching (Thompson et al., 2023). The Physical Education Director's previous background as a physical education teacher facilitated close work with physical education teachers (demonstrated by the 10 % increase in MVPA during lesson time). However, with limited time, he did not focus on principals, who have the power to change physical education scheduling (whereas physical education teachers do not). In addition, despite being the “Physical Education Director,” his technical position as a “teacher on special assignment” did not give him the authority over principals that someone with a true director position might hold. This suggests that principals' physical education-related priorities (i.e., ensuring 100 min are scheduled) may not change in the absence of larger support for the subject (i.e., funding for physical education teachers) or support from higher up within the district.

While encouraging that MVPA increased across all schools, there are potential reasons why MVPA increased non-differentially between groups. First, all schools received $1000/year as incentive for participation, which schools could have applied towards independent teacher trainings and/or equipment. Second, there was likely intervention contamination. Because the Physical Education Director was obligated under his general job description to work with all schools, control school physical education teachers also benefited from his coaching if they attended district-wide professional development opportunities or reached out directly for assistance.

A strength of this research is the randomized design in a real-world public-school setting. However, the restriction to a single district and a relatively small sample limits generalizability. Further, study randomization did not include presence of a full-time credentialed physical education teacher in the school (which was unknown/uncertain at randomization). In addition, PEAFC was based on a fully funded model initially implemented by NYCDOE. In the current district, PEAFC implementation was unfunded, which limited the physical education Director's ability to adopt NYCDOE's best practices. In addition, we did not have strong data on intervention dose (i.e. number of interactions with the school principal, number of times a physical education teacher received coaching), which would have aided in the analyses. Results could be different in schools with more district-level support for physical education. Nonetheless, the results support existing literature indicating that increasing compliance with physical activity mandates in schools is challenging without financial support (Boles et al., 2011).

Randomizing elementary schools to receive PEAFC from the Physical Education Director over two years did not result in increased compliance with California state physical education law nor in increased student lesson time MVPA. Full-time credentialed physical education teachers were employed at all schools which met the state physical education mandate, while over two-thirds of schools not meeting the mandate did not have full-time credentialed physical education teachers. In the absence of having enough physical education teachers to deliver all state-mandated physical education minutes, and without funding for district personnel to support school-level implementation, PEAFC may be limited in its potential to increase compliance with physical education mandates. Additional randomized studies powered to examine the impact of PEAFC with and without physical education teachers are necessary. Funding credentialed physical education teachers in the elementary school setting has repeatedly been shown to be the most effective way to support schools in complying with state physical education laws.

## Disclosure of funding and conflicts of interest

This work was funded by the National Heart Lung and Blood Institute (NHLBI), Grant 1K01HL151805. The funder had no role in the study design, collection, analysis, and interpretation of data or in writing the manuscript. The authors have no conflicts of interest to disclose.

## CRediT authorship contribution statement

**Hannah R. Thompson:** Writing – original draft, Supervision, Project administration, Methodology, Investigation, Funding acquisition, Formal analysis, Conceptualization. **Caroline Nguyen:** Writing – review & editing, Project administration, Methodology, Data curation. **Thomas L. McKenzie:** Writing – review & editing, Methodology. **David A. Dzewaltowski:** Writing – review & editing, Methodology, Conceptualization. **Kristine A. Madsen:** Writing – review & editing, Methodology, Conceptualization.

## Funding

This work was funded by the National Heart Lung and Blood Institute (NHLBI), Grant 1K01HL151805. The funder had no role in the study design, collection, analysis, and interpretation of data or in writing the manuscript.

**Trial Registration:**ClinicalTrials.gov Identifier: NCT05509803

## Declaration of competing interest

The authors declare that they have no known competing financial interests or personal relationships that could have appeared to influence the work reported in this paper.

## Data Availability

Data will be made available on request.
